# Simultaneous Determination of Mineral Nutrients and Toxic Metals in *M. stenopetala* from Southern Ethiopia: A Comparative Study of Three Cultivating Areas Using MP-AES

**DOI:** 10.1155/2024/8981995

**Published:** 2024-01-05

**Authors:** Ashenafi Shemnsa, Wondimeneh Dubale Adane, Merid Tessema, Endale Tesfaye, Gizaw Tesfaye

**Affiliations:** ^1^Department of Chemistry, Addis Ababa University, P.O. Box 1176, Addis Ababa, Ethiopia; ^2^Department of Chemistry, Gambella University, P.O. Box 126, Gambella, Ethiopia; ^3^Department of Chemistry, Fitche College of Teachers Education, P.O. Box 260, Fitche, Ethiopia

## Abstract

In this study, for the first time, the levels of thirteen micro- and macromineral nutrients in the leaves, seeds, and supportive soil of *Moringa stenopetala* (*M. stenopetala*) were simultaneously determined using microwave plasma atomic emission spectroscopy (MP-AES). The samples were collected during the arid season, in 2019 from the three main *M. stenopetala* growing areas in southern Ethiopia (Chano Mile Kebele, Nechisar Kebele, and Konso Special Woreda). A novel digestion method for leaf and seed samples was developed using an optimized acid mixture (2.5 : 0.75 : 0.5 of HNO_3_, HClO_4_, and H_2_O_2_) at 240°C for 2 hrs and 30 min, resulting in clear and colorless solutions. The method makes the digestion process more efficient by minimizing the reagent volume, reducing digestion temperature and time, and simplifying the overall procedure. The efficiency of the optimized procedure was validated by spiking experiments, and the percentage recovery ranged between 94 and 110%. Under optimized experimental conditions, higher concentrations of essential minerals (K, Na, Ca, and Mg) were observed in the plant leaf and seed samples from the three areas. In addition, significant amounts of trace elements (Fe, Mn, Zn, and Cu) were also found. Importantly, no traces of the toxic elements (Cd and Pb) were detected in any of the analyzed samples, suggesting that the leaves and seeds of *M. stenopetala* are valuable sources of both micro- and macromineral nutrients and are safe from toxic metals. From a dietary perspective, the seed contains almost comparable concentrations of minerals as the leaves. As a result, the seeds of *M. stenopetala* can serve as an alternative source of minerals and play a role in overcoming the current global food crisis, particularly in the dry season. Analysis of variance at a 95% confidence level revealed significant differences in the levels of all mineral nutrients between the three sample means except K, Ca, Co, and Cu. Generally, the developed method includes an innovative digestion procedure that minimizes reagent consumption, operates at lower temperatures, and requires shorter digestion times, thereby optimizing resource utilization and maintaining analytical accuracy. Notably, the absence of toxic elements in the MP-AES procedure highlights the safety and reliability of *M. stenopetala* leaves and seeds as valuable, contamination-free sources of essential nutrients.

## 1. Introduction

Moringa, a family of *Moringaceae*, grows throughout the tropics. It is a versatile tree of significant economic importance as it has vital nutritional and medicinal uses. The genus *Moringa* consists of 13 species, of which *Moringa oleifera (M. oleifera)* and *Moringa stenopetala (M. stenopetala)* are the most widespread species and share numerous common characteristics [1, 2]. *M. oleifera* is native to the sub-Himalayan districts of northern India and is commonly known as the “horseradish” or “drumstick” tree. *M. oleifera* varies from *M. stenopetala* in growth pattern, foliage, blossoms, and seedpod characteristics [1]. *M. stenopetala* is native to southern Ethiopia, where it is known as Haleko or Shiferaw. Its primary purpose of cultivation is for its edible leaves, which are consumed as vegetables [3]. The plant has high drought resistance, and its leaves have an extremely favorable nutrient composition. In addition, the leaves exhibit antioxidant properties and possess therapeutic effects against various human diseases due to the presence of the biomolecule rutin, which has antioxidant and antidiabetic properties [4, 5]. The leaves of *M. stenopetala* are an important food source for millions of people in southern Ethiopia, especially during the dry season. It is a rapid-growing plant that is effortlessly developed in marginal areas and less fertile soils in dry environments and could therefore serve as a reliable source of food and income for numerous societies [5, 6].

Plants play a significant role as providers of dietary macro and trace elements that are vital for human health. The elements have a vital importance in numerous bodily functions. The primary means of obtaining essential and nonessential elements for the body is through the intake of food [7]. In the past, people relied on consuming natural foods such as plants, seeds, nuts, and leaves to fulfill their nutritional requirements, making them the primary source of essential macro and trace elements in the diet [8]. Many developing nations are marked by rapid population growth combined with declining agricultural productivity. This led to a quest for an alternative source of high-quality yet affordable food, drawing inspiration from indigenous knowledge [9]. Consequently, the utilization of plant leaves, such as those of *M. stenopetala*, is becoming more widespread as they have a significant impact on addressing the difficulties of diminishing food supplies while also offering nutritious dietary options. The growing fascination with functional foods has additionally led to a rise in the intake of organic food sources such as *M. stenopetala*.

Essential elements are crucial and required by living organisms to activate enzymes and generate hormones. Some of these elements are part of organic molecules involved in the growth and maintenance of life processes, while others play significant roles in the bone structure [8, 10]. Some trace elements are important for biological processes, and their deficiency or surplus can disrupt the normal functions of the body [11]. For instance, a trace amount of iron is important for transporting oxygen in the body and promoting strength [12]. Manganese is used in fat and carbohydrate metabolism as well as in blood sugar regulation and helps the body build connective tissues and bones and aids in blood clotting [13]. Zinc acts as a cofactor for certain enzymes, also involved in metabolism and cell growth, and plays an essential role in various other biological processes. It is also crucial for maintaining a robust immune system and protection against infection [14]. However, certain elements can be hazardous if the highest daily intake exceeds over an extended period. The Joint Panel of Experts on Food Additives of the FAO, World Health Organization, and European Union (Regulation no. 1881/2006) have established maximum levels for Pb (0.3 mg kg^−1^) and Cd (0.2 mg kg^−1^) in plant leaves and fresh herbs [8]. WHO has established the highest permissible levels for toxic elements in herbal plants as 5, 10, 0.3, 2.0, and 0.2 mg kg^−1^ for As, Pb, Cd, Cr, and Hg, respectively [15]. The European Food Safety Authority considers the prevention and management of Pb pollution as crucial due to its detrimental effects on the human body and excessive concentration in the environment [16].

The levels of toxic metals in the leaves of edible plants often exceed safe limits, especially when grown in contaminated areas [17]. The quality of edible leaves and seeds is mainly influenced by the environment in which they grow [18]. Therefore, many literature reports focused on the determination of heavy metal content in the leaves and roots of *M. stenopetala* [19–21]. However, in some studies, the concentrations of toxic metals in the leaves and seeds of *M. stenopetala* have been reported [22, 23]. Soil is not only the growing medium but also one of the sources of metal pollution in edible plants [17]. To the best of our knowledge, there is no literature report on the mineral content in the leaves, seeds, and the corresponding soil of the three main *M. stenopetala* cultivating areas in the Gamo Zone of southern Ethiopia (Chano Mile and Nechisar Kebeles) and Konso Special Woreda. Therefore, it is important to assess the levels of essential and nonessential metals in the leaves, seeds, and growing soil of *M. stenopetala* to ensure the quality and safety of the plant and to assess possible contamination.

The development of suitable analytical methods to determine essential and nonessential elements in various edible plants and vegetables is of great interest. Numerous analytical techniques, including flame atomic absorption spectrometry (FAAS), X-ray fluorescence spectrometry (XRF), graphite furnace atomic absorption spectrometry (GFAAS), inductively coupled plasma mass spectrometry (ICP-MS), and atomic fluorescence spectrometry (AFS), have been used to quantify elements in different matrices [24]. In the analysis of plant seeds, roots, leaves, and vegetables, spectrometric techniques such as atomic absorption spectrometry (AAS), microwave plasma atomic emission spectroscopy (MP-AES), inductively coupled plasma optical emission spectrometry (ICP-OES), and inductively coupled plasma mass spectrometry (ICP-MS) are commonly used [25–27]. Compared to AAS and AES, MP-AES is more sensitive for the rapid and simultaneous determination of essential and nonessential elements at trace or ultratrace levels in complex samples [28].

The innovative aspect of this study lies in its pioneering attempt to provide fundamental insights into the concentration of essential and nonessential elements in the leaves, seeds, and supportive soil of *M. Stenopetala* from major production regions in the Gamo Zone, southern Ethiopia, specifically Chano Mile and Nechisar Kebeles, as well as Konso Special Woreda. The method employed in this study is characterized by the development of an acid digestion-based multielement analysis using MP-AES. This cutting-edge approach allows the simultaneous determination of various elements, including K, Na, Ca, Mg, Fe, Co, Ni, Mn, Zn, Cr, Cu, Cd, and Pb. The advantages of this method include its ability to provide a comprehensive analysis of multiple elements in a single run, offering efficiency and time-saving. Additionally, the utilization of MP-AES ensures precision and accuracy in the determination of element concentrations. However, it is essential to acknowledge potential limitations and disadvantages of the method, such as the sensitivity to sample matrix effects or the need for careful calibration. The current study not only contributes essential data on mineral content in different parts of *M. Stenopetala* but also aims to address the gap in analytical techniques for such complex determinations. By comparing the levels of identified metals in the plant with existing literature values, this research provides a robust foundation for future studies in the field, emphasizing the significance of its innovative methodology and potential implications for understanding the ecological and nutritional aspects of *M. Stenopetala*.

## 2. Materials and Methods

### 2.1. Sampling Area Description

The research was carried out in the Gamo Zone (Chano Mile and Nechisar Kebeles) and Konso Special Woreda in the SNNPR during the dry season in 2019. Gamo Zone is situated in the south-central part of Ethiopia, with coordinates of 6°14′60.00″ N latitude and 37°00′0.00″ E longitude. It is located 502 km to the south of Addis Ababa. The elevation in this area ranges from 600 to 4207 m above sea level, covering an area of 6735 km^2^. The temperature varies between 10 and 25°C, while the annual rainfall ranges from 200 to 2000 mm. The Gamo Zone is known for its two lakes, Abaya and Chamo [29]. Konso Special Woreda, on the other hand, is located at 5° 20′ 25.6164″ N latitude and 37° 26′ 19.6404″ E longitude, approximately 607.2 km to the south of Addis Ababa. The elevation in this region ranges from 501 to 2000 m above sea level, covering a land area of 2,016.24 km^2^. The Woreda is characterized by 70% low altitude and 30% tropical midaltitude. The average annual temperature in Konso Special Woreda is recorded between 17.6 and 27.5°C, with an average annual rainfall ranging from 601 to 1200 mm [30].

### 2.2. Chemicals and Reagents

HClO_4_ (70%), HNO_3_ (69–72%) (BDH Laboratory Supplies, Anala®, Poole, England), and H_2_O_2_ (30%) (Scharlau Chemie, European Union, Spain) were used for the digestion of the samples. Lanthanum (III) nitrate hexahydrate (BDH Chemicals Ltd., Poole, England) was used to minimize the interference of Ca^2+^ and Mg^2+^ ions. Stock solutions of 1000 mg L^−1^ of the metals K, Na, Ca, Mg, Fe, Co, Ni, Mn, Zn, Cr, Cu, Cd, and Pb (BDH Chemicals Ltd. Spectrosol, Poole, England) were used for preparing calibration standards and spiking experiments. Distilled water was used to dilute the samples, intermediate, and working metal standard solutions before analysis and to rinse the glassware.

### 2.3. Instruments and Apparatus

A digital analytical balance (Scitech) with an accuracy of 0.0001 g was used to weigh the samples. An electronic blender (Moulinex, France) was used to grind and homogenize the samples. 100 mL round-bottom flasks fitted with a reflux condenser in a Kjeldahl apparatus (UK) were used to digest the samples. A microwave plasma atomic emission spectrometer (Agilent Technologies, Model 4200, USA) was used for the analysis of K, Na, Ca, Mg, Fe, Co, Ni, Mn, Zn, Cr, Cu, Cd, and Pb.

### 2.4. Sample Collection

During the main harvest season (dry season commonly from October to December), leaf, seed, and soil samples of *M. stenopetala* were collected from the three main moringa growing areas of Gamo Zone (Chano Mile and Nechisar Kebeles) and Konso Special Woreda. Ten sites from each area were selected for leaf and seed sampling. Again, eight farmers were randomly selected from a single area. At least five *M. stenopetala* plants were then randomly selected from each farm for leaf and seed sampling. Soil samples were collected systematically at all locations under each sampled *M. stenopetala* plant at a radius of 100 cm and a depth of 50 cm. As the investigation focused on the possible uptake of metals by the plant, soil samples were collected from the entire area penetrated by the root system. Finally, the samples were thoroughly homogenized to form representative leaf, seed, and soil samples of *M. stenopetala* for each plot.

### 2.5. Sample Preparation

#### 2.5.1. Preparation of Leaf Samples

Moringa leaves, comprising a blend of both young and mature leaves (exceeding 100 in total), were thoroughly cleaned to eliminate external impurities from their surfaces. The leaves were washed with tap water, distilled water, and deionized water for 30, 15, and 10 min, respectively, and then dried in a laboratory under direct sunlight for weeks. The dried leaves were finally pulverized with an electric motor and milled into a fine powder, of which 25 g was used for subsequent analysis.

#### 2.5.2. Preparation of Seed Samples

Over 200 seed samples, including both young and mature specimens, were subjected to a thorough cleaning process. This included careful removal of debris, soil, twigs, and foliage as well as the exclusion of immature, overripe, and damaged pods to ensure the selection of the highest quality seeds. The husks were dried in a clean area and exposed to direct sunlight. The seeds were removed from the shell after a drying process that lasted for more than four weeks. The dried seeds were eventually ground into a fine powder, and 25 g of this was taken for investigation.

#### 2.5.3. Preparation of Soil Samples

Visible plant debris was removed from the collected soil samples; the sample was then air-dried and homogenized. The dried soil samples were ground and sieved through a 2 mm sieve. The total amount of soil samples collected from each district was over 500 g, of which 25 g was used for analysis.

### 2.6. Optimization of Digestion Procedure

The primary prerequisite for sample preparation for analysis is the establishment of optimum conditions for digestion. The optimum conditions are those that require the least amount of reagent volume, the shortest reflux time, clear solution, and simplicity [7, 31]. As a result, we carefully selected optimal digestion conditions for the leaf and seed samples, which utilize minimal reagent volume and a shorter digestion time, resulting in a clear and colorless solution at a lower temperature. Thus, several experiments were conducted with 0.5 g of leaf and seed powder samples using acid mixtures (HNO_3_, HClO_4_, and H_2_O_2_) to produce a clear and colorless solution. Similarly, other experimental parameters, such as digestion time and digestion temperature, were carefully examined to obtain the optimal digestion values (Tables 1–3). As shown in the tables, the mixture of acids 2.5 : 0.75 : 0.5 (2.5 mL HNO_3_, 0.75 mL HClO_4_, and 0.5 mL H_2_O_2_), a digestion time of 2:30 hrs, and a digestion temperature of 240°C were selected to be the optimal experimental conditions for the digestion of 0.5 g of leaf and seed samples.

### 2.7. Digestion of Leaf, Seed, and Soil Samples

Under the optimized conditions, 0.5 g of moringa leaf powder was transferred to 250 mL round-bottom flasks. 10 mL of the acid mixture (2.5 : 0.7 : 0.5 (v/v)) was added, and the mixture was digested in a Kjeldahl apparatus for two and a half hours at a temperature of 240°C. Thereafter, the digested mixture was allowed to cool to room temperature and then filtered through a filter paper (Whatman. 42). The digestion was performed in triplicate, and parallel to the digestion of the samples, a reagent blank was also digested, keeping all digestion parameters the same. Six blanks were digested for leaf samples. When determining Ca^2+^ and Mg^2+^, to avoid chemical interference, a solution of 0.1% LaCl_3_·7H_2_O in deionized water was used. The same procedure was followed for the digestion of seed samples under the optimized conditions. The method EPA 3050B (EPA 3050B, 1996) with a very slight modification was used for the digestion of soil samples (briefly discussed in Supplementary Material).

### 2.8. Preparation of Standard Solutions

The working standard solutions for each metal were prepared from a 1000 mg L^−1^ standard stock solution of the elements K, Na, Ca, Mg, Fe, Co, Ni, Mn, Zn, Cr, Cu, Cd, and Pb in 5% HNO_3_. Intermediate standard solutions of 10 mg L^−1^ were prepared in 100 mL volumetric flasks from the stock standard solution. Since the linear ranges of the calibration curves differed, different concentrations of calibration standards were used for different metals. Four working standards were freshly prepared for each metal from the intermediate standards by dilution with deionized water. Three replicate determinations were made for each element, and the same analytical procedures were used for blank solutions.

### 2.9. Determination of Metals in Leaf, Seed, and Soil Samples

Following the calibration of the instrument, the levels of specific metals in the samples were measured using MP-AES. Three separate analyses were conducted for each sample. K, Na, Ca, Mg, Fe, Co, Ni, Mn, Zn, Cr, Cu, Cd, and Pb were measured in the emission/concentration mode after accurately calibrating the instrument using a calibration blank and four working calibration standard solutions. The measurement of metals in the digested blank solution was also carried out simultaneously with the samples, maintaining consistent parameters and employing the same procedure.

### 2.10. LOD and LOQ

The limit of detection (LOD) is the smallest value at which an analyte can be measured with some certainty. The limit of quantification (LOQ) is the lowest concentration of an analyte that can be quantitatively measured with a specified level of accuracy and precision [32, 33]. The LOD and LOQ values of the mineral nutrients were determined using seven duplicate blanks. The LOD was obtained by multiplying the pooled standard deviation of the blank (*δ*_blank_) by three (LOD = 3*δ*_blank_, where *δ* = standard deviation of the blank). The LOQ was calculated as ten times the standard deviation of the blank solution (LOQ = 10*δ*_blank_). The LOD of the elements studied (Table 4) was lower than the analyte concentrations obtained.

### 2.11. Method Validation

Limit of detection (LOD), LOQ, precision, accuracy, and linearity were examined as the analytical parameters for determining the elements. The accuracy of the method was assessed by spiking samples with a known concentration of the analyte standard solution. Therefore, the leaf, seed, and soil samples were spiked with different volumes of stock solutions. K, Na, Ca, Mg, Fe, Co, and Zn samples (each 0.5 g) were spiked with 0.75 mL of the respective stock solution. On the other hand, for Mn, Cr, Ni, Cu, Cd, and Pb, a larger volume of 2.75 mL of the corresponding stock solution was added to each 0.5 g of the sample. The validated method was employed to digest and measure the spiked and nonspiked samples under the optimized conditions using MP-AES (instrument operating parameters are given in Table S1). The percentage recovery for the leaf, seed, and soil samples is displayed in Table 5, and it varies from 94 to 110%, which falls within the acceptable range and shows the validity of the optimized procedure.

### 2.12. Statistical Analysis

The preparation of calibration curves and data analysis was carried out using Origin 6.0 software. To validate and compare the average values of the metals across various sampling sites, a one-way ANOVA was employed. Pearson's correlation coefficient was utilized to ascertain the extent of positive or negative correlation among the metals.

## 3. Results and Discussion

### 3.1. Concentration of Metals in *M. stenopetala* Leaf, Seed, and Soil Samples Collected from the Three Areas

In the study, a total of thirteen elements were investigated using MP-AES. The most abundant mineral nutrients in the *M. stenopetala* plant parts and the supportive soil of the three areas were K, Ca, Mg, Na, and Fe. K, Ca, and Mg concentrations were higher in the leaves, seeds, and growing soil than in the mean total concentrations. Furthermore, we have considered Cr and Ni as micronutrients because they are essential for human health in small amounts. WHO has set the maximum permissible concentration of Cr and Ni in drinking water as 50 *μ*g/L and 70 *μ*g/L, respectively. Cr and Ni can cause a variety of negative health effects, including cancer, skin irritation, and respiratory and kidney problems [7].

#### 3.1.1. Concentrations of Mineral Nutrients in Leaf Samples

**Figure 1:**
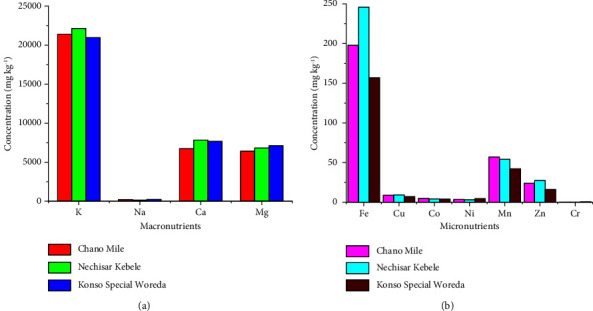
The concentrations of (a) macro- and (b) micromineral nutrients in the leaves of *M. stenopetala* from the three areas.

As shown in Table 6, there is a significant difference in macro- and micronutrient contents within and between leaf samples of *M. stenopetala*. The most abundant element among the macronutrients is K, followed by Ca, Mg, and Na (Figure 1(a)). The main reason for the high potassium content in the leaves of *M. stenopetala* is the fact that nutrients such as nitrogen, phosphorus, potassium, sulfur, and magnesium are highly mobile in the plant tissue and move from older plant tissues to newer ones. Another factor contributing to the higher concentration of K, Mg, and Ca is that these elements are frequently used for plant growth and development [7]. In addition, the abundance of calcium-bearing minerals in soil and water, which are normally abundant and readily absorbed by plants, also contributes to elevated Ca levels [34]. In contrast, among the investigated micronutrients, the Fe content of the leaves was dominant, followed by Mn, Zn, Cu, Co, Ni, and Cr (Figure 1(b)). In general, it can be concluded from the levels of all metals in the leaves of *M. stenopetala* that the concentrations of macro- and micronutrients in the three areas follow a similar trend. Dado et al. [35] and Yetesha et al. [36] reported a similar trend in mineral concentrations in the leaves of *Pentas schimperiana* and various parts of pumpkin, respectively.

In general, the relative abundance of mineral nutrients in the leaves of *M. stenopetala* follows this sequence: K > Ca > Mg > Na > Fe > Mn > Zn > Cu > Co > Ni > Cr. The toxic metals Cd and Pb were not detected in the leaves of *M. stenopetala* from the three districts. This finding suggests that there is no contamination from toxic industrial waste in the sampling areas. Furthermore, the absence of harmful metals suggests that commercial fertilizers and herbicides are likely not used in *M. stenopetala* plantations in the vicinity. Moreover, Cd and Pb offer no nutritional benefit to humans; their minimal concentrations are appreciable. As a result, despite the potential health risks brought by the toxic metals, *M. stenopetala* leaves from the Chano Mile, Nechisar Kebele, and Konso Special Woreda are safe. The distribution patterns of each metal in the leaves of *M. stenopetala* are consistent in the three areas. A one-way analysis of variance revealed that the mean concentrations of all identified metals in the leaves of the three districts differed significantly at a 95% confidence level. The varying concentrations of metals could be attributed to differences in the age and variety of the sampled *M. stenopetala* plants [37].

#### 3.1.2. Concentrations of Mineral Nutrients in Seed Samples

**Figure 2:**
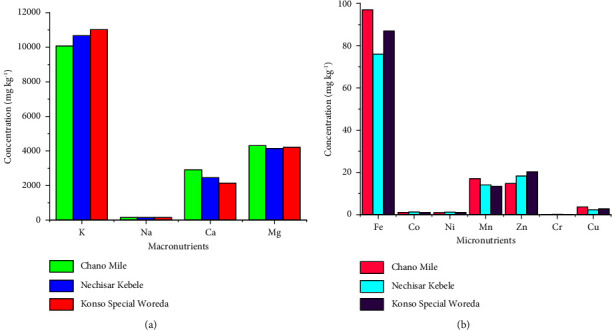
The concentrations of (a) macro- and (b) micromineral nutrients in the seeds of *M. stenopetala* from the three areas.

The concentration of K in the seed samples was the highest among the macromineral nutrients (Table 6) and the highest of all mineral nutrients analyzed, followed by Mg, Ca, and Na. Macromineral nutrients detected in *M. stenopetala* seeds had the following concentration trend: K > Mg > Ca > Na (Figure 2(a)). Among the sample sites, the highest concentration of K was found in *M. stenopetala* seeds from Konso Special Woreda, followed by Nechisar Kebele and Chano Mile. Mg concentration was the highest in seed samples from Chano Mile, followed by Konso Special Woreda and Nechisar Kebele. The concentration levels of Ca found in the seed samples from the three sites were as follows (highest to lowest): Chano Mile, Nechisar Kebele, and Konso Special Woreda. This indicates that the Ca content in the Chano Mile seed sample is significantly higher (*p* < 0.05) than that of Nechisar Kebele and Konso Special Woreda. The Nechisar Kebele seed sample has the highest Na content of the three sampling areas. The Na values in the seed samples from Nechisar Kebele and Konso Special Woreda show no significant differences at a 95% confidence level. As shown in Figure 2(b), Fe is the most abundant micronutrient in seed samples, followed by Mn, Zn, Cu, Co, Ni, and Cr. Table 6 also shows that the concentration ranges of all micronutrients are almost closer in the three sample areas. The increased Fe concentration is a consequence of its higher concentration in the supportive soil [31, 38]. It was found that the concentrations of Cd and Pb are below the detection limit, and the plant has a very limited ability to accumulate these toxic metals in its tissues.

#### 3.1.3. Concentrations of Mineral Nutrients in Soil Samples

**Figure 3:**
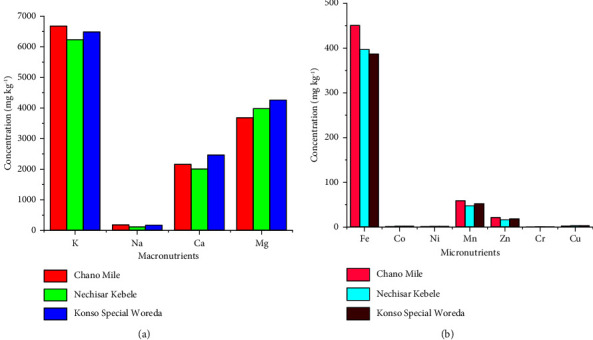
The concentrations of (a) macro- and (b) micromineral nutrients in the supportive soil of *M. stenopetala* plants from the three areas.

As indicated in Table 6, the levels of macro- and micromineral nutrients detected in the soils of *M. stenopetala* farms differed significantly from one area to another. Among the macromineral nutrients investigated, K exhibited the highest concentration, followed by Mg, Ca, and Na. In terms of micromineral nutrients, Fe was found to be the most abundant element in the soil samples, followed by Mn, Zn, Cu, Co, Ni, and Cr. The soil of the Nechisar Kebele root zone had the least amount of K, whereas the Chano Mile site had the highest concentration. Konso Special Woreda showed the highest Ca content, while Nechisar Kebele had the lowest concentration. Similarly, the soil sample from Konso Special Woreda had the highest Mg concentration, while Chano Mile had the lowest (Figure 3(a)). In the soil sample from Chano Mile, Fe was the micromineral nutrient with the highest concentration, followed by Nechisar Kebele and Konso Special Woreda (Figure 3(b)). On the other hand, the concentrations of Mn and Zn follow the same trend, with the highest value being at Chano Mile, followed by Konso Special Woreda and Nechisar Kebele. For Cu, the highest content was recorded in Nechisar Kebele, followed by Konso Special Woreda and Chano Mile. As in the leaf and seed samples, Cd and Pb were not detected in all soils studied in the three areas. The results indicate that the agricultural soils of the *M. stenopetala* plant are free from heavy metal contamination.

### 3.2. Distribution Patterns of Metals in the Samples

Plants can absorb mineral nutrients through diverse and complex biochemical pathways. The uptake varies depending on the ability of plants to take up mineral nutrients from the soil, the availability of nutrients in soluble and absorbable forms, and the presence of specific nutrients at the site. Increasing industrialization and pollution of the biosphere are the main reasons for the variation in soil concentrations of vital minerals and toxic metals [39]. Various fertilizers, pesticides, and other chemicals are used on agricultural land, which become sources of soil pollution and make the quality of agricultural land unfit for human and animal lives. The distribution and accumulation of mineral nutrients in the *M. stenopetala* plant reflect the mineral composition of the soil and the environment in which the plants grow [40]. Therefore, the mineral content of *M. stenopetala* varies greatly depending on the geographic location, use of fertilizers, and other factors such as irrigation water. If we compare the concentration of mineral nutrients by sample location in leaf samples, the trend can be presented as follows: Nechisar Kebele > Chano Mile > Konso Special Woreda. Konso Special Woreda > Nechisar Kebele > Chano Mile in Mg content. Nechisar Kebele > Chano Mile > Konso Special Woreda in Fe content. Chano Mile > Nechisar Kebele > Konso Special Woreda in Mn content. The concentration variations of Co and Ni are not very pronounced at the three sites, suggesting that the distribution of these metals is invariant compared to other elements. The variation of Fe by sample location was the highest among the micromineral nutrients, with concentrations ranging from 687 to 451 mg kg^−1^, which is below the WHO/FAO permissible limit of 5000 mg kg^−1^. The variation of Cu by sample location was the lowest, with concentrations ranging from 2.64 to 3.61 mg kg^−1^, which is also below the WHO guideline value of 4 mg kg^−1^ for agricultural soils [41, 42], as shown in Table 6.

In general, plants absorb what is present in the soil medium. As a result, the mineral nutrients are also absorbed and accumulated in the roots, stems, fruits, grains, and leaves of the plant. Ultimately, these minerals can be transferred to humans through the food chain. The absorption process is strongly influenced by soil nutrient content, soil pH, the presence of other binders, the ionic strength of the solution in the soil, and the presence of other competing nutrients [43]. Therefore, the occurrence of these studied mineral nutrients in the leaves and seeds of *M. stenopetala* can be attributed to their increased concentration in the soil.

### 3.3. Comparison of the Mineral Nutrients of This Work with Literature Values and FAO/WHO Guidelines

The mineral concentrations obtained in this research were compared with studies by other scientists. Different scientists have conducted diverse studies on the leaves of *M. stenopetala* (mainly edible part). However, no comprehensive studies have been conducted on the mineral nutrient composition of the leaves of *M. stenopetala* from the Nechisar Kebele, Chano Mile, and Konso Special Woreda areas.

As illustrated in Table 7, the mineral nutrients obtained from the leaf samples of *M. stenopetala* in this study are in accordance (with a few exceptions) with other investigations from the same and different countries. For example, the level of K identified in this research was less than that documented by Solomon and Kusse [44] and Sodamode [47]. In comparison with other literature findings, Mg is present at elevated concentrations in the present study. In comparison to the findings of Solomon and Kusse [44] and Sodamode et al. [47], Ca is present in higher concentrations but is lower than those reported by Debebe and Eyobel [45]. In contrast to the findings of Solomon and Kusse [44] and Debebe and Eyobel [45], Fe is present in higher concentrations but is lower than those reported by Nkuba and Mohammed [46] and Sodamode et al. [47]. In comparison with Solomon and Kusse [44] and Nkuba and Mohammed [46], Mn is present in higher concentrations but is lower than those reported by Debebe and Eyobel [45] and Sodamode et al. [47]. The Zn concentration determined in this study also agrees well with values from other researchers such as Debebe and Eyobel [45] and Nkuba and Mohammed [46] but is lower than those reported by Solomon and Kusse [44] and Sodamode et al. [47]. As shown in Table 7, the concentrations of Cd and Pb were very low compared with some literature reports. The variations between the results are likely attributed to differences in soil composition, genetic diversity, and environmental conditions. Furthermore, external factors such as municipal waste, fertilizers, irrigation practices, pollution, and climate variability may also affect the rate at which plants accumulate metals [48]. To conclude, the concentrations of analyzed metals in *M. stenopetala* leaves were found to be within safe limits, as stipulated by the FAO/WHO guidelines [49]. These results contribute to our understanding of the nutritional safety of *M. stenopetala* leaves, supporting their suitability for consumption within established regulatory standards.

### 3.4. Daily Intake of Mineral Nutrients from *M. stenopetala* Leaves

The daily consumption of minerals from *M. stenopetala* leaves was calculated based on the assumption that an average adult consumes an average of 200 g of dried moringa leaves per day. The quantities of minerals a person acquires from the leaves of *M. stenopetala* are listed in Table 8. Among the trace mineral nutrients, the amount of Na that a person can consume is lower than the recommended daily values, indicating that only the leaves of *M. stenopetala* cannot serve as a reliable source of Na for daily requirements. Therefore, additional Na needs to be obtained from other sources. For other trace minerals, the leaves of *M. stenopetala* can provide sufficient nutrition. Among the essential mineral nutrients, the amount of Zn that humans can absorb is also lower than the necessary amount. Hence, supplemental nutrition is necessary for this nutrient. However, the levels of Fe and Mn are abundant, making the leaves of *M. stenopetala* the ideal food source for the communities in the three regions and the entire country, especially during the dry season. The levels of Cd and Pb are below detectable limits in *M. stenopetala* leaves from the three regions, suggesting that consuming these leaves is generally safe from the risks associated with Cd and Pb exposure. However, it is important to note that Pb and Cd are cumulative toxins with the potential to accumulate in the body over time, even at low levels.

### 3.5. Statistical Analysis

#### 3.5.1. Analysis of Variance (ANOVA)

In this study, leaf, seed, and supportive soil samples were collected from the three main *M. stenopetala* growing areas in Ethiopia, and the mineral content of each sample was analyzed using MP-AES. During sample preparation and analysis, a number of random errors can occur with each aliquot and replicate measurement. The variation in the sample mean of the analyte was tested using a one-way ANOVA [51] to investigate whether the variation was due to the experimental procedure or due to sample heterogeneity.  Table 9 shows that at the 95% confidence level, there are significant differences in the means of all analytes examined except K, Ca, Co, and Cu. The significant difference between the mean values of the samples can be attributed to the diversity of soil mineral composition or pH, which determines the extent of mineral assimilation by plants [7].

#### 3.5.2. Pearson's Correlation Coefficient of Metals

The correlation between the mineral nutrients in *M. stenopetala* leaves and soil samples was examined using Pearson's correlation matrices to determine the correlation coefficients (Tables S2A and S2B). For the majority of the mineral nutrients obtained, except Mn and Cu in the leaves of *M. stenopetala* and Co, Zn, and Cr in the soil samples, the correlation was significant at the 95% confidence level. In addition, Ni showed a negative correlation with almost all elements in both samples and a weak association with the others. A highly positive correlation indicates a strong relationship between the nutrients, possibly derived from natural sources or the environment, or similarity in chemical properties [52]. The correlation between Mg and Cu in plants and Co and Mn in soil was negative.

As shown in Table 10, the correlation coefficients (*r*) between the mineral nutrients in the leaves of *M. stenopetala* and the soil samples collected from the three areas were computed for each metal individually. The outcomes indicated that there was a strong correlation between K, Na, Ca, Mg, Fe, Ni, and Cu in the leaf and soil samples, and a weak correlation between Cr, Zn, Mn, and Co was observed in the samples. Strong correlations between soil mineral content and plant tissue were anticipated, as plants take up nutrients from the soil through their roots. The weak correlation observed for some metals could be attributed to the fact that some nutrients, even when present in high concentrations in the soil, may be taken in by the plant to a lesser extent as they may not be accessible in a soluble form for absorption by the plant.

The results of our analytical endeavors unveil a nuanced portrait of the micro- and macromineral composition of *M. stenopetala*, laying a foundation for profound insights into its nutritional significance. The methodological innovation inherent in our study, employing MP-AES, represents a leap forward in the simultaneous determination of thirteen essential minerals in *M. stenopetala* leaves, seeds, and soil. This approach not only streamlines the digestion process with an optimized acid mixture but also enhances precision by minimizing reagent volumes and reducing digestion time and temperature. The observed elevated concentrations of essential minerals, including K, Na, Ca, and Mg, underscore the potential of *M. stenopetala* as a valuable source of vital nutrients. Remarkably, the absence of toxic elements such as Cd and Pb fortifies the safety profile of *M. stenopetala* leaves and seeds. These findings contribute not only to the burgeoning field of plant nutrition but also position our developed method as an innovative and efficient tool for comprehensive mineral analysis. As we chart the course forward, these revelations guide the trajectory of our research proposal, prompting further exploration into the multifaceted dimensions of *M. stenopetala*'s nutritional attributes and its potential impact on mitigating dietary deficiencies and bolstering food security in arid regions.

## 4. Conclusion

In this comprehensive study, we employed MP-AES coupled with the acid digestion method to analyze *M. stenopetala* leaf, seed, and supportive soil samples from Chano Mile Kebele, Nechisar Kebele, and Konso Special Woreda in southern Ethiopia. While the optimized digestion procedure exhibited robustness, as evidenced by excellent percentage recoveries ranging from 94 to 110% in the spiking experiment, it is imperative to acknowledge certain limitations inherent in the method. Potential constraints may include sensitivity variations for specific minerals or potential interference in the detection process. Moreover, the analysis results revealed higher concentrations of both microminerals (K, Na, Ca, and Mg) and macrominerals (Fe, Mn, Zn, and Cu) in plant leaf and seed samples from the three areas, contributing valuable insights to the nutritional values of *M. stenopetala*. However, it is crucial to note that the study has limitations. Differences in soil physical and chemical composition, fertilizer and pesticide usage, agroclimatic conditions, and harvesting mechanisms among the three regions may introduce variability in the observed mineral concentrations. These inherent variations underscore the complexity of the agroecosystem and emphasize the need for cautious interpretation. Notably, the absence of toxic elements Cd and Pb in any of the analyzed samples confirms the safety of *M. stenopetala* leaves and seeds. Despite these strengths, it is essential to recognize that the study's generalizability may be influenced by the specific characteristics of the sampled regions and may not fully capture the variability present in other geographical areas. A one-way ANOVA at a 95% confidence level revealed significant differences in the content of all mineral nutrients between the three sample means, excluding K, Ca, Co, and Cu. While this emphasizes the need for a region-specific approach to interpret the results, the concentrations of micro- and macromineral nutrients in *M. stenopetala* leaves and seeds were generally found to be within safety limits, supporting their suitability for consumption as a healthy food source.

## Figures and Tables

**Table 1:** Optimization of acid mixture volume for the digestion of 0.5 g of leaf and seed samples at a constant temperature and digestion time.

Volume ratio (HNO_3_ : HClO_4_ : H_2_O_2_)	Total volume (mL)	Temp. (°C)	Digestion time (hrs)	Observations
2 : 1 : 1	4	300	3	Slightly green color
2.5 : 1 : 1	4.5	300	3	Slightly green color
3.5 : 1 : 1	5.5	300	3	Light yellow
4 : 1 : 1	5	300	3	Colorless and turbid
2.5 : 0.5 : 1	4	300	3	Colorless and turbid
2.5 : 0.75 : 1	4.25	300	3	Colorless with suspension
2.5 : 1 : 1	4.5	300	3	Colorless with suspension
2.5 : 1.25 : 1	4.75	300	3	Clear and colorless
2.5 : 0.75 : 0.5	3.75	300	3	^*∗*^Clear and colorless

^*∗*^The optimal acid mixture volume.

**Table 2:** Optimization of digestion time for 0.5 g of leaf and seed samples at a constant acid mixture volume and digestion temperature.

Volume ratio (HNO_3_ : HClO_4_ : H_2_O_2_)	Total volume (mL)	Temp. (°C)	Digestion time (hrs)	Observations
2.5 : 0.75 : 0.5	3.75	300	1:00	Yellow solution
2.5 : 0.75 : 0.5	3.75	300	1:30	Light yellow
2.5 : 0.75 : 0.5	3.75	300	2:00	Colorless and turbid
2.5 : 0.75 : 0.5	3.75	300	2:30	^*∗*^Clear and colorless
2.5 : 0.75 : 0.5	3.75	300	3:00	Clear and colorless

^*∗*^The optimal digestion time.

**Table 3:** Optimization of digestion temperature for 0.5 g of leaf and seed samples at a constant acid mixture volume and digestion time.

Volume ratio (HNO_3_ : HClO_4_ : H_2_O_2_)	Total volume (mL)	Temp. (°C)	Digestion time (hrs)	Observations
2.5 : 0.75 : 0.5	3.75	60	2:30	Light yellow
2.5 : 0.75 : 0.5	3.75	120	2:30	Colorless and turbid
2.5 : 0.75 : 0.5	3.75	180	2:30	Colorless with suspension
2.5 : 0.75 : 0.5	3.75	240	2:30	^*∗*^Clear and colorless
2.5 : 0.75 : 0.5	3.75	280	2:30	Clear and colorless

^*∗*^The optimal digestion temperature.

**Table 4:** Wavelength, LOD, LOQ, correlation coefficient, and calibration curve equation to determine the metals by MP-AES.

Metals	Wavelength (nm)	LOD (mg L^−1^)	LOQ (mg L^−1^)	Correlation curve	*r* ^2^
K	766.5	0.036	0.12	*y* = 0.718*x* – 4.72 × 10^−6^	0.9996
Na	589.0	0.034	0.114	*y* = 0.689*x* + 1.46 × 10^−3^	0.9981
Ca	393.4	0.026	0.087	*y* = 0.346*x* – 6.25 × 10^−5^	0.9953
Mg	285.2	0.024	0.08	*y* = 0.177*x* + 2.58 × 10^−3^	0.9994
Fe	372.0	0.008	0.027	*y* = 0.322*x* + 8.45 × 10^−5^	0.9987
Co	240.7	0.006	0.02	*y* = 0.541*x* + 5.46 × 10^−5^	0.9954
Ni	352.5	0.005	0.017	*y* = 0.451*x* + 5.35 × 10^−5^	0.9976
Mn	403.1	0.005	0.017	*y* = 0.368*x* – 3.17 × 10^−4^	0.9988
Zn	213.9	0.001	0.004	*y* = 0.226*x* – 2.31 × 10^−5^	0.9992
Cr	425.4	0.006	0.02	*y* = 0.212*x* + 3.16 × 10^−4^	0.9994
Cu	324.8	0.003	0.01	*y* = 0.223*x* – 5.68 × 10^−5^	0.9952
Cd	228.8	0.001	0.004	*y* = 0.541*x* + 4.37 × 10^−4^	0.9962
Pb	283.2	0.005	0.017	*y* = 0.541*x* + 4.37 × 10^−4^	0.9997

**Table 5:** Recovery analysis results for *M. stenopetala* leaf, seed, and soil samples.

Metals	Leaf				Seed				Soil			
	Unspiked (mg kg^−1^)	Added (mg kg^−1^)	Obtained conc. (mg kg^−1^)	Recovery (%)	Unspiked (mg kg^−1^)	Added (mg kg^−1^)	Obtained conc. (mg kg^−1^)	Recovery (%)	Unspiked (mg kg^−1^)	Added (mg kg^−1^)	Obtained conc. (mg kg^−1^)	Recovery (%)
K	22142	5600	27542	96.43	10071	2800	12771	96.43	6487	3546	10063	100.9
Na	153	85	231	91.77	65.6	42	106	96.20	2174	1520	3654	97.37
Ca	7842	4328	12190	100.5	2912	2164	5086	100.5	2469	1520	3919	95.40
Mg	6842	4116	10998	100.9	4312	2058	6380	100.5	4259	2425	6634	97.94
Fe	246	120	358	93.34	97	60	155	96.67	387	125	511	99.20
Co	4.35	2.76	7.05	97.83	1.14	1.38	2.49	97.83	2.12	1.52	2.25	100.9
Ni	3.21	2.18	5.38	99.55	1.06	1.09	2.08	93.58	1.86	1.52	3.32	96.06
Mn	54.28	30.92	84.8	98.71	17.14	15.46	32.7	100.7	52.36	25.75	76.01	91.85
Zn	27.64	25.42	53.16	100.4	14.82	12.71	27.45	99.38	18.65	6.25	24.5	93.60
Cr	0.32	1.12	1.41	97.33	0.11	0.56	0.66	98.22	0.87	1.12	1.91	92.86
Cu	9.42	4.54	13.94	99.56	3.71	2.27	5.89	96.04	3.51	1.12	4.57	94.65
Cd	ND^*∗*^	1.34	1.28	95.52	ND^*∗*^	0.65	0.61	93.85	ND^*∗*^	0.65	0.63	96.72
Pb	ND^*∗*^	1.01	0.98	97.03	ND^*∗*^	0.5	0.49	98.00	ND^*∗*^	0.5	0.48	96.00

^*∗*^ND = not detected.

**Table 6:** Mean concentration (mean ± SD, *n* = 3, mg kg^−1^ dry weight basis) of each metal in the three areas.

Elements		Chano Mile			Nechisar Kebele			Konso Special Woreda		
		L_CM_	Sd_CM_	So_CM_	L_NK_	Sd_NK_	So_NK_	L_KS_	Sd_KS_	So_KS_
Mean ± SD (mg kg^−1^)	K	21390 ± 100	10070 ± 80	6680 ± 30	22140 ± 70	10680 ± 40	6230 ± 34	20990 ± 90	11020 ± 72	6490 ± 50
	Na	213.0 ± 2.1	160.6 ± 2.4	187 ± 4.5	153.4 ± 2.8	175.4 ± 2.6	123 ± 3.3	245 ± 3.4	157.75 ± 3.2	174 ± 4.1
	Ca	6749 ± 16	2912 ± 24	2164 ± 17	7842 ± 21	2458 ± 17	2014 ± 25	7695 ± 21	2146 ± 11	2469 ± 20
	Mg	6425 ± 53	4312 ± 36	3684 ± 39	6842 ± 23	4152 ± 30	3984 ± 26	7128 ± 36	4215 ± 28	4259 ± 22
	Fe	198 ± 4	97 ± 3	451 ± 5.5	246 ± 2.8	76 ± 1.9	397 ± 3.5	157 ± 2.2	87 ± 1.2	387 ± 2.9
	Co	4.98 ± 0.75	1.14 ± 0.06	1.65 ± 0.05	4.35 ± 0.06	1.29 ± 0.05	2.31 ± 0.03	4.12 ± 0.07	1.09 ± 0.06	2.12 ± 0.02
	Ni	3.7 ± 0.1	1.06 ± 0.01	1.47 ± 0.01	3.21 ± 0.05	1.25 ± 0.04	1.94 ± 0.02	4.78 ± 0.06	1.11 ± 0.03	1.86 ± 0.05
	Mn	57.28 ± 1.51	17.14 ± 1.5	59.23 ± 2.1	54.28 ± 1.1	14.12 ± 1.2	47.5 ± 1.9	42.2 ± 1.2	13.46 ± 1.1	52.36 ± 1.2
	Zn	24.12 ± 0.20	14.82 ± 0.4	21.65 ± 0.12	27.64 ± 0.24	18.36 ± 0.11	16.24 ± 0.15	16.18 ± 0.68	20.36 ± 0.41	18.65 ± 0.19
	Cr	0.22 ± 0.06	0.11 ± 0.02	0.42 ± 0.04	0.32 ± 0.02	0.24 ± 0.01	0.96 ± 0.05	0.62 ± 0.03	0.18 ± 0.01	0.87 ± 0.02
	Cu	8.96 ± 0.05	3.71 ± 0.04	2.64 ± 0.01	9.42 ± 0.04	2.31 ± 0.01	3.26 ± 0.02	7.13 ± 0.04	2.84 ± 0.02	3.51 ± 0.03
	Cd	<LOD	<LOD	<LOD	<LOD	<LOD	<LOD	<LOD	<LOD	<LOD
	Pb	<LOD	<LOD	<LOD	<LOD	<LOD	<LOD	<LOD	<LOD	<LOD

L = leaf; Sd = seed; So = soil; CM = Chano Mile; NK = Nechisar Kebele; and KS = Konso Special Woreda.

**Table 7:** Comparison of the means of the investigated minerals in *M. stenopetala* leaves with literature values.

Source	Means of the examined minerals (mg kg^−1^)										References
	K	Mg	Ca	Fe	Mn	Zn	Cu	Ni	Cd	Pb	
Ethiopia	31797.5	—	6167	63.02	26.83	44.09	3.58	—	0.05	—	[44]
Ethiopia	10865	4321.5	18914.5	81.49	74.57	26.59	4.5	—	—	—	[45]
Tanzania	14541	5058.13	—	309.57	73.47	19.88	4.35	2.25	—	0.35	[46]
Nigeria	2320.0	6770	7230	870	25.20	54.80	5.50	—	—	—	[47]
Ethiopia	21508	6798.34	7428.67	200.34	51.25	22.65	8.51	3.88	—	—	This work

**Table 8:** Comparison of daily intake of mineral nutrients from *M. stenopetala* leaves with recommended daily intake and tolerable upper limit of daily intake.

Elements	Concentration in leaf (mg kg^−1^)	Amount of nutrients per 200 g of leaf consumed	Recommended daily intake [7, 50]	Upper tolerance limit [7, 50]
K	20987–22142	4200–4426.2	4700 mg	NE
Na	153–245	30.6–49	1500 mg	2300 mg/day
Ca	7842–6749	1568.4–1349.8	1000–1200 mg	2500 mg/day
Mg	7128–6425	1425.6–1285	320–420 mg	750 mg/day
Fe	157–246	31.4–49.2	10–15 mg	45 mg/day
Co	4.12–4.98	0.85–0.1	5–40 *μ*g/day	0.25 mg/day
Mn	42.2–57.3	8.24–11.4	1.8–2.3 mg	11 mg/day
Zn	16.2–27.6	3.32–5.53	10–15 mg	40 mg/day
Cr	0.22–0.62	0.04–0.1	25–35 *μ*g	120 *μ*g/day
Cu	7.13–9.42	1.79–1.88	0.9–2 mg	10 mg/day

NE = not established.

**Table 9:** Analysis of variance between and within *M. stenopetala* leaf samples at a 95% confidence level.

Elements	Comparison	SD (mg kg^−1^)	D_f_	F_cal_	F_crit_	Remark
K	BS	93	4	2.29	2.54	NSD
	WS	85	40			

Na	BS	2.05	4	38.91	2.54	SDs
	WS	2.72	40			

Ca	BS	15.6	4	1.61	2.54	NSD
	WS	19.4	40			

Mg	BS	53.1	4	54.18	2.54	SDs
	WS	37.2	40			

Fe	BS	3.37	4	44.58	2.54	SDs
	WS	2.79	40			

Co	BS	0.75	4	1.87	2.54	NSD
	WS	0.3	40			

Ni	BS	0.08	4	67.48	2.54	SDs
	WS	0.07	40			

Mn	BS	1.15	4	49.27	2.54	SDs
	WS	1.32	40			

Zn	BS	0.19	4	59.64	2.54	SDs
	WS	0.37	40			

Cr	BS	0.06	4	61.45	2.54	SDs
	WS	0.04	40			

Cu	BS	0.05	4	2.41	2.54	NSD
	WS	0.05	40			

BS = between samples, WS = within samples, NSD = no significant difference, SDs = significant difference, SD = standard deviation, D_f_ = degree of freedom, F_cal_ = Fcalculated, and F_crit_ = Fcritical.

**Table 10:** Pearson's correlation between mineral nutrients in *M. stenopetala* leaves and soil samples (*n* = 4).

Elements	K	Na	Ca	Mg	Fe	Co	Ni	Mn	Zn	Cr	Cu
*r*	0.869	0.842	0.997	0.797	0.898	0.437	0.887	0.442	0.551	0.584	0.921

## Data Availability

The data used to support the findings of the study are included within the article.

## Supplementary Materials

Supplementary Materials Table S1: MP-AES operating parameters. Table S2A: Pearson's correlation for *M. stenopetala* leaf samples. Table S2B: Pearson's correlation for supportive soil samples.
