# Supplementary material for: Simultaneous Determination of Mineral Nutrients and Toxic Metals in M. stenopetala from Southern Ethiopia: A Comparative Study of Three Cultivating Areas Using MP-AES
Source: J Anal Methods Chem. 2024 Jan 5;2024:8981995. doi: 10.1155/2024/8981995 (PMC10787013; doi:10.1155/2024/8981995)
Supplement: Supplementary Materials — Table S1: MP-AES operating parameters. Table S2A: Pearson's correlation for M. stenopetala leaf samples. Table S2B: Pearson's correlation for supportive soil samples. [file 8981995.f1.docx]

**Supplementary Materials**

**Simultaneous Determination of Mineral Nutrients and Toxic Metals in *M. Stenopetala* from Southern Ethiopia: A Comparative Study of Three Cultivating Areas Using MP-AES**

Ashenafi Shemnsa **^[^**^1^**^]^,** Wondimeneh Dubale Adane ^[1]^, Merid Tessema*^[1]^, Endale Tesfaye ^[2]^, and Gizaw Tesfaye ^[3]^

**^[1]^** Department of Chemistry, Addis Ababa University, P. O. Box 1176, Addis Ababa, Ethiopia

**^[2]^** Department of Chemistry, Gambella University, P. O. Box 126, Gambella, Ethiopia

**^[3]^** Department of Chemistry, Fitche College of Teachers Education, P. O. Box 260, Fitche, Ethiopia

*Corresponding author. E-mail addresses: [tessmer2265@yahoo.com](mailto:tessmer2265@yahoo.com), [merid.tessema@aau.edu.et](mailto:merid.tessema@aau.edu.et)

**1. Digestion of soil samples**

The method EPA 3050B (EPA 3050B, 1996), with a very minor modification, was used for the digestion of soil samples. The dried and sieved soil sample (0.5 g) was placed in a digestion vessel, and 10 ml of 1:1 HNO_3_ and H_2_O (deionized) were added. The sample was heated to 110 °C and refluxed for 25 min. It was then allowed to cool, and 5 ml of concentrated HNO_3_ was added and refluxed for 120 min. After 2 hrs, due to the appearance of brown fumes, the digestion was stopped by adding 5 ml of conc. HNO_3_ twice, at interval of 10 min, until no brown fumes were released from the sample, indicating completion of the reaction with HNO_3_. The sample was cooled, and 3 mL of deionized water and 5 mL of H_2_O_2_ (30%) were added. The sample was heated for 90 min until excessive foaming disappeared. After cooling, 10 ml of concentrated HCl was added to the sample and heated to 95°C for 25 min. Finally, the digest was allowed to cool, filtered through Whatman #42 filter paper, and the resulting clear, light yellow solution was made up to 50 mL with deionized water. Reagent blanks were also prepared and digested using the same procedure as for the soil sample.

**Table S1.** MP-AES operating parameters

| **Plasma condition** | |
| --- | --- |
| Power of magnetron output | 1 kW |
| **Gas flow** | |
| Plasma gas flow-nitrogen | 20 l/min |
| Intermediate flow-nitrogen | 1.5 l/min |
| Pre-optics protection gas- air | 25 l/min |
| Nebulizer pressure | 140-240 kPa (optimized for each element) |
| Spray chamber | Double-pass glass cyclonic |
| Torch | Quartz torch |
| Plasma viewing | Axial |
| **Acquisition parameters** | |
| Sample uptake delay | 12 s |
| Stabilization time | 20 s |
| Read time | 5 s |
| No. of replicates | 5 |
| Back ground correction | Auto of FLIC (fast linear interference correction) |
| Optical system | Czerny-Turner design monochromater with 600 mm focal length and fixed entrance slit |
| Detector | Back-thinned solid state CCD detector (532x128 pixels) |

**Table S2A.** Pearson’s correlation for *M. stenopetala* leaves samples

|  | K | Na | Ca | Mg | Fe | Co | Ni | Mn | Zn | Cr | Cu |
| --- | --- | --- | --- | --- | --- | --- | --- | --- | --- | --- | --- |
| K | 1.00 |  |  |  |  |  |  |  |  |  |  |
| Na | 0.85 | 1.00 |  |  |  |  |  |  |  |  |  |
| Ca | 0.89 | 0.68 | 1.00 |  |  |  |  |  |  |  |  |
| Mg | 0.67 | 0.98 | 0.86 | 1.00 |  |  |  |  |  |  |  |
| Fe | 0.99 | 0.82 | 0.77 | 0.93 | 1.00 |  |  |  |  |  |  |
| Co | 0.84 | 0.76 | 0.88 | 0.35 | 0.99 | 1.00 |  |  |  |  |  |
| Ni | -0.73 | -0.82 | -0.76 | -0.91 | -0.82 | 0.98 | 1.00 |  |  |  |  |
| Mn | 0.24 | 0.45 | 0.2 | 0.35 | 0.22 | 0.29 | 0.41 | 1.00 |  |  |  |
| Zn | 0.87 | 0.99 | 0.84 | 0.76 | 0.59 | 0.64 | 0.75 | 0.77 | 1.00 |  |  |
| Cr | 0.77 | 0.88 | 0.78 | 0.86 | 0.64 | 0.99 | 0.76 | 0.99 | 0.83 | 1.00 |  |
| Cu | 0.32 | 0.13 | 0.39 | 0.47 | 0.45 | 0.25 | 0.12 | 0.16 | 0.35 | 0.2 | 1.00 |

**Table S2B**. Pearson’s correlation for supportive soil samples

|  | K | Na | Ca | Mg | Fe | Co | Ni | Mn | Zn | Cr | Cu |
| --- | --- | --- | --- | --- | --- | --- | --- | --- | --- | --- | --- |
| K | 1.00 |  |  |  |  |  |  |  |  |  |  |
| Na | 0.78 | 1.00 |  |  |  |  |  |  |  |  |  |
| Ca | 0.82 | 0.94 | 1.00 |  |  |  |  |  |  |  |  |
| Mg | 0.68 | 0.72 | 0.84 | 1.00 |  |  |  |  |  |  |  |
| Fe | 0.86 | 0.99 | 0.76 | 0.68 | 1.00 |  |  |  |  |  |  |
| Co | 0.23 | 0.41 | 0.12 | 0.36 | 0.28 | 1.00 |  |  |  |  |  |
| Ni | -0.76 | -0.68 | -0.99 | -0.88 | -0.68 | -0.78 | 1.00 |  |  |  |  |
| Mn | 0.87 | 0.65 | 0.59 | 0.84 | 0.92 | 0.75 | 0.84 | 1.00 |  |  |  |
| Zn | 0.25 | 0.36 | 0.12 | 0.46 | 0.34 | 0.28 | 0.19 | 0.44 | 1.00 |  |  |
| Cr | 0.24 | 0.43 | 0.16 | 0.27 | 0.34 | 0.33 | 0.41 | 0.46 | 0.28 | 1.00 |  |
| Cu | 0.78 | 0.69 | 0.88 | 0.94 | 0.63 | 0.76 | 0.84 | 0.94 | 0.99 | 0.75 | 1.00 |
